# Comprehensive Magnetic Resonance Imaging Relaxometry of Gadolinium‐Based Contrast Agents: A Systematic Study of Transmetallation and Transchelation Processes With Zinc Ions and Heparin

**DOI:** 10.1002/cmdc.202501096

**Published:** 2026-03-26

**Authors:** Patrick Werner, Leif Schröder

**Affiliations:** ^1^ Translational Molecular Imaging Deutsches Krebsforschungszentrum Heidelberg Germany; ^2^ Department of Radiology Charité – Universitätsmedizin Berlin Berlin Germany; ^3^ Department of Physics and Astronomy Ruprecht‐Karls Universität Heidelberg Heidelberg Germany; ^4^ German Cancer Consortium (DKTK) Partner Site Heidelberg Heidelberg Germany

**Keywords:** gadolinium, gadolinium‐based contrast agent, glycosaminoglycans, relaxometry, transchelation

## Abstract

Gadolinium‐based contrast agents (GBCAs) are essential in medical imaging, but concerns remain about their long‐term safety. An increasing number of studies indicate that gadolinium can accumulate in human tissues. The initial step is transmetallation, whereby endogenous ions displace Gd(III) ions from its chelate. Subsequently, a transchelation process allows ion binding to macromolecules, such as glycosaminoglycans (GAGs), and tissue deposits may form. However, the clinical impact and potential connection to gadolinium deposition disease remain uncertain and require further research. In this study, magnetic resonance imaging relaxometry was employed to investigate the molecular interactions of eight clinically relevant GBCAs with Zn(II) ions and GAGs. Taking advantage of the characteristic relaxivity changes of Gd(III) ions in different environments enabled detection and quantification of free Gd(III) ions, as well as of the macromolecule‐bound species. The investigation revealed distinct relaxivity patterns of Gd‐accessible water that are specific for each GBCA, highlighting differences in intrinsic stability when challenged by competing ions or alternative chelators. Evaluation of transitions between equilibrium states enables comparative assessment of reaction kinetics for deeper insights into the observables of GBCA behavior. Thus, relaxometry emerges as a robust analytical platform, offering valuable guidance for improving existing contrast agents and designing safer, more stable alternatives.

## Graphical abstract

Water proton relaxometry studies reveal unique signatures for different classes of gadolinium‐based magnetic resonance imaging contrast agents (GBCAs) undergoing transchelation with glycosaminoglycans. While linear nonionic GBCAs exhibit significantly reduced thermodynamic stability during transchelation, they show higher stability during transmetallation than their dianionic counterparts.

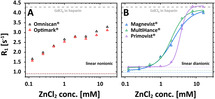

## 1 Introduction

Contrast‐enhanced magnetic resonance imaging (MRI) is a well‐established diagnostic procedure with a wide range of applications in clinical settings. The technique is routinely used for the identification of disease and pathological alterations. Its central importance as a diagnostic tool is due to its ability to easily depict changes in soft tissues and, in contrast to other imaging techniques such as computed tomography (CT), the abstention from utilizing ionizing radiation. Gadolinium‐based contrast agents (GBCAs) are administered to provide additional morphological and functional information compared to unenhanced MR images [1, 2].

Gadolinium, a rare earth and heavy metal used in these agents, is bound by a chelator molecule to prevent toxic effects [3, 4]. Regarding the structure of the chelator, GBCAs can be categorized into different groups. The ligands used to bind gadolinium rely on two different approaches to coordinate this central ion. Linear chelators wrap the Gd(III) ions in a polyamino carboxylic acid backbone that does not completely enclose the metal ion. In contrast, macrocyclic ligands completely encompass the central ion. This structural difference results in a hierarchy of stability, with ionic macrocyclic chelates being the most stable, followed by nonionic macrocyclic, then ionic linear, and finally nonionic linear chelates being the least stable [5]. This ranking relates to the assumed safety profile of different types of GBCAs in clinical applications. With over 460 million doses administered worldwide since 1988, GBCAs are indispensable in clinical daily routine. In 2016, ≈200,000 MRI scans utilizing GBCAs were conducted daily in the European Union and the United States. In Germany alone, over 32,000 MRI scans are performed on a daily basis. Between 33% and 50% of all clinical examinations were performed with intravenous injection of GBCAs [6, 7, 8] with ≈1.1 g of Gd per MRI scan [9]. This corresponded to a use of ca. 50 t per year in 2019 [10]. It is important to note that the amount of GBCAs administered is highly variable and may be modified based on each individual patient and the progression of the specific disease [11]. This is intended to illustrate that every patient is exposed to different degrees of risk and, depending on the individual diagnosis, is exposed to GBCAs with varying frequency.

Nevertheless, after the injection in the patient, these GBCAs rapidly distribute throughout the body and are primarily eliminated by passive glomerular filtration (>95%) [1]. In individuals with normal renal function, the plasma half‐life of GBCAs is ≈1.5 h [12]. However, in people with impaired renal filtration rates, the plasma half‐life can exceed a day and comes with great variability (34.3 ± 22.9 h) [13]. This prolonged half‐life increases the availability of GBCAs in the blood and may result in increased release of free Gd. It is important to note that the exact half‐life may vary depending on the specific GBCA and the degree of renal insufficiency, which as a result may enable increased interactions with endogenous substances.

These findings have led to a re‐evaluation of the overall safety profiles of various GBCAs, with significant concerns about the long‐term consequences of Gd deposition in the body, particularly in the case of repeated administration. The major symptoms, summarized under the term Gd deposition disease (GDD), are peripheral neuropathic pain, joint stiffness, muscle spasms, a buzzing sensation, fatigue, and distal extremity and skin thickening [14, 15].

One of the most significant concerns associated especially with linear GBCAs is the transmetallation step, whereby Gd(III) ions are displaced from their chelator molecule by competing metal ions [16, 17, 18, 19, 20], including zinc (Zn^2+^), iron (Fe^3+^), calcium (Ca^2+^), and copper (Cu^2+^) [21]. This displacement can result in the release of toxic free Gd(III) ions into the body. Evidence of this process has been observed in the form of elevated zinc levels in urine following the administration of GBCAs [9]. Furthermore, endogenous chelators, such as glycosaminoglycans (GAGs), are suspected to bind gadolinium [19, 20, 22], thereby contributing to its deposition in tissues. Precipitation as Gd phosphate is also discussed, but this would not explain clinically observed hyperintense signals in *T*_1_‐weighted images.

To address these issues, a number of regulatory measures have been introduced in recent years to minimize the risks associated with GBCA administrations. The European Medicines Agency (EMA) has prohibited the use of specific linear GBCAs, whereas in Japan, linear ligands may only be employed as an alternative in the absence of macrocyclic agents, due to clinical considerations [9, 21]. However, in countries such as the USA and China, which account for a significant proportion of the global population, there have been no amendments to the legal regulations governing the use of GBCAs. The US Food and Drug Administration (FDA) has only issued warnings regarding the potential risks associated with the use of these agents in patients with renal dysfunction. Furthermore, the possibility of Gd retention has been highlighted, although its clinical significance remains uncertain [11].

Nevertheless, it is currently still under debate whether GBCAs themselves, their transformation products, or the free gadolinium released from transmetallation represent a health risk. However, the process of transmetallation, particularly involving metals such as zinc, is hypothesized to increase the potential toxicity of GBCAs and further research is required to gain a better understanding of the long‐term effects and mechanisms of GBCA/Gd retention in the human body [23, 24]. One important aspect is the identification of observables that can be linked to clinical procedures. Relaxometry is easy to translate and scalable for a larger number of samples. We thus hypothesized that the relaxometry profiles along the transmetallation and transchelation progress may reveal common patterns that reflect on the GBCA subgroups and may reflect on the structure‐vulnerability relationship with and without the presence of a macromolecular scavenger of released Gd(III) ions.

The aim of this study is to use the NMR relaxometry technique published in 2021 [19] to explore and quantify the combined effects of competing ions and macromolecular chelators on the stability of eight worldwide commonly used GBCAs. This covers evaluation of how effectively endogenous chelators can sequester Gd(III) ions and how much zinc is required to induce the transmetallation process. The primary focus is on comparing the steady‐state relaxivity without addressing the kinetics of GBCA transmetallation and transchelation, and aspect that will be investigated in more detail in future work. Specifically, we investigate heparin for its (Gd) ion‐binding capacity and quantify the complete transchelation process induced by zinc from all GBCAs to heparin.

## 2 Results and Discussion

### 2.1 Relaxivity Determinations Under Stable Conditions

**FIGURE 1:**
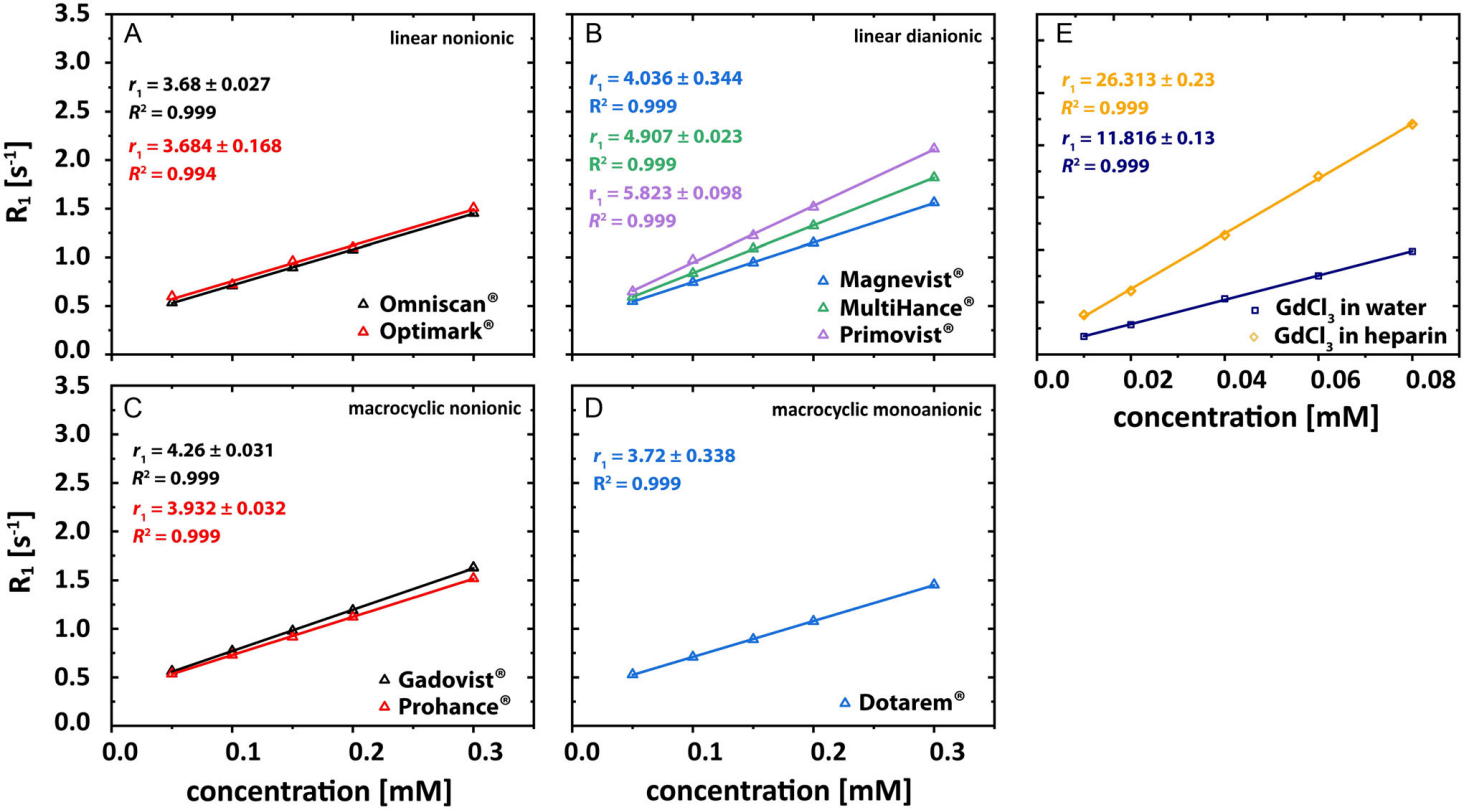
Relaxivity plots for: (A) Omniscan (black triangles), Optimark (red triangles); (B) Magnevist (purple triangles), MultiHance (green triangles), Primovist (blue triangles); (C) Gadovist (black triangles), ProHance (red triangles), and (D) Dotarem (blue triangles). The relaxivity plot for the used polysaccharide is shown in (E) for heparin (yellow circles) in H_2_O. The relaxivity values are given by the slopes of the obtained linear fits. Note the different concentration ranges for subplot (E).

We measured the relaxivities (*r*_1_) of all individual compounds used in well‐defined model solutions under different conditions, determining them in both nanopure water and aqueous heparin solutions. All measured relaxivities for the used GBCAs and all other relaxivity results are summarized in Table S1. For a better visualization, Figure 1 provides an overview of the individual relaxivities of the used GBCAs and GdCl_3_ with and without heparin.

The relaxivity of GdCl_3_ (*r*_1 _≈ 11.8 ± 0.1 s^−1^ mM^−1^) is ≈2–3 times higher than for GBCAs in nanopure water (Table S1). However, when comparing the GdCl_3_ *r*_1_ value in 100 μM heparin solution (*r*_1 _≈ 26.3 ± 0.2 s^−1^ mM^−1^) it can be seen that it is 2.5‐fold higher than the value in nanopure water. The differences in the observed relaxivities of the Gd(III) ion‐containing components are the prerequisite for the subsequent quantifications of different processes occurring in GBCA destabilization. The relaxivity of ZnCl_2_ in nanopure water was about fourfold lower than all other components utilized in the test solutions. By taking a look at the values for the pure polysaccharide in nanopure water, it can be seen that heparin has a relaxivity of *r*_1 _≈ 0.01 ± 0.3 s^−1^ mM^−1^.

### 2.2 GBCA Transmetallation and Gd(III) Transchelation Processes

**FIGURE 2:**
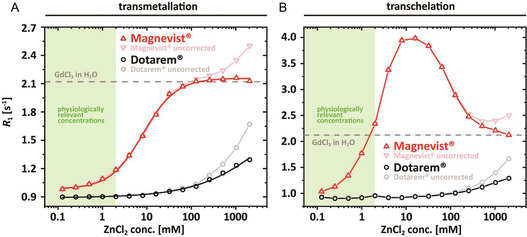
Uncorrected *R*_1_ values as a function of the ZnCl_2_ concentration for Magnevist (light red triangles), Dotarem (light black circles) and corrected *R*_1_ values taking the ZnCl_2_ contribution into account (bold symbols) in (A) nanopure water and (B) 100 μM heparin‐containing aqueous solution. All curves start at *R*_1_ values that match the expected relaxation of 150 μM of the corresponding GBCA in nanopure water or heparin. For increasing ZnCl_2_ concentration, *R*_1_ of all curves increases. Uncorrected *R*_1_ values overlap initially but are significantly higher for ZnCl_2_ concentrations >100 mM. The values for Magnevist in nanopure water approach a new plateau value of *R*_1_ ≈ 2.16 ± 0.009 s^−1^ at zinc concentrations >100 mM. In heparin solution, Magnevist reaches a transient plateau at *R*_1_ ≈ 3.98 ± 0.1 s^−1^ (ZnCl_2_ > 8 mM) with a subsequent decrease of the *R*_1_ value to the level of 150 μM free GdCl_3_ in nanopure water.

ZnCl_2_ was used to induce the release of Gd(III) ions from GBCAs for transmetallation and transchelation experiments. The relaxation caused by ZnCl_2_ alone with increasing amounts of divalent ions must be corrected to eliminate unwanted contributions in the quantification. This is illustrated in Figure 2, which shows the corrected *R*_1_ values as a function of ZnCl_2_ concentrations between 0 and 2048 mM for 150 μM Magnevist (red triangles) and Dotarem (black circles) in nanopure water (A) and in 100 μM heparin‐containing aqueous solution (B). The light and dark colors represent the values with and without correction for the contribution of ZnCl_2_, respectively.

All curves start at a level where they match the expected relaxivity of 150 μM GBCA in nanopure water or in heparin solution. For transmetallation, *R*_1_ increases with increasing ZnCl_2_ concentrations, and the values for Magnevist asymptotically approach a plateau value of *R*_1_ ≈ 2.16 ± 0.009 s^−1^ in nanopure water for [ZnCl_2_] > 100 mM. For transchelation in heparin‐containing solutions, a transient plateau (*R*_1_ ≈ 3.98 ± 0.1 s^−1^) is reached after the addition of 8 mM ZnCl_2_. A subsequent decrease of the corrected relaxation rates to the level of 150 μM GdCl_3_ in nanopure water is recorded for [ZnCl_2_] > 16 mM. This data goes beyond the original application published in 2021 [19] and illustrates that the same observed *R*_1_ can occur around 2 mM but also at 1000‐fold higher Zn^2+^ concentrations when apparently all of the once heparin‐bound Gd is also transmetalled in the GAG and exists as aqueous ion only. The *R*_1_ value must thus be seen in the context of subsequent competitions with Zn(II) ions, where the rising flank in Figure 2B represents a situation with partially intact GBCA but the same *R*_1_ on the falling flank occurs after full Gd release from the parent chelator.

The measured profile illustrates how Zn(II) ions and Gd(III) ions compete for the GAG ion‐binding sites. The expected maximum *R*_1_ = 4 s^−1^ for 150 µm Gd(III) in heparin is in fact reached for 10 mM Zn^2+^, but soon after followed by a loss in *R*_1_, for [Zn^2+^] > 16 mM, the available Gd(III) ions are stepwise released from the GAG and finally appear to be free in solution with all heparin binding sites being occupied by excessive Zn(II) ions. The *R*_1_ values of the uncorrected data points increase again for divalent ion concentrations greater than 512 mM, thus demonstrating once more the need for correction and identification of the new equilibrium. The uncorrected and corrected results for Dotarem demon‐strate a consistent increase with rising ZnCl_2_ concentrations that represents the late and incomplete transmetallation compared to the linear GBCA. Given the low relaxivity of ZnCl_2_ (*r*_1,__*ZnCl*__2_ < 10^−4^ s^−1^ mM^−1^), the corrected and uncorrected curves differ only for ZnCl_2_ concentrations exceeding 100 mM. All subsequent figures in this study show data sets that have been corrected for the impact of ZnCl_2_ to ensure accuracy and precision.

**FIGURE 3:**
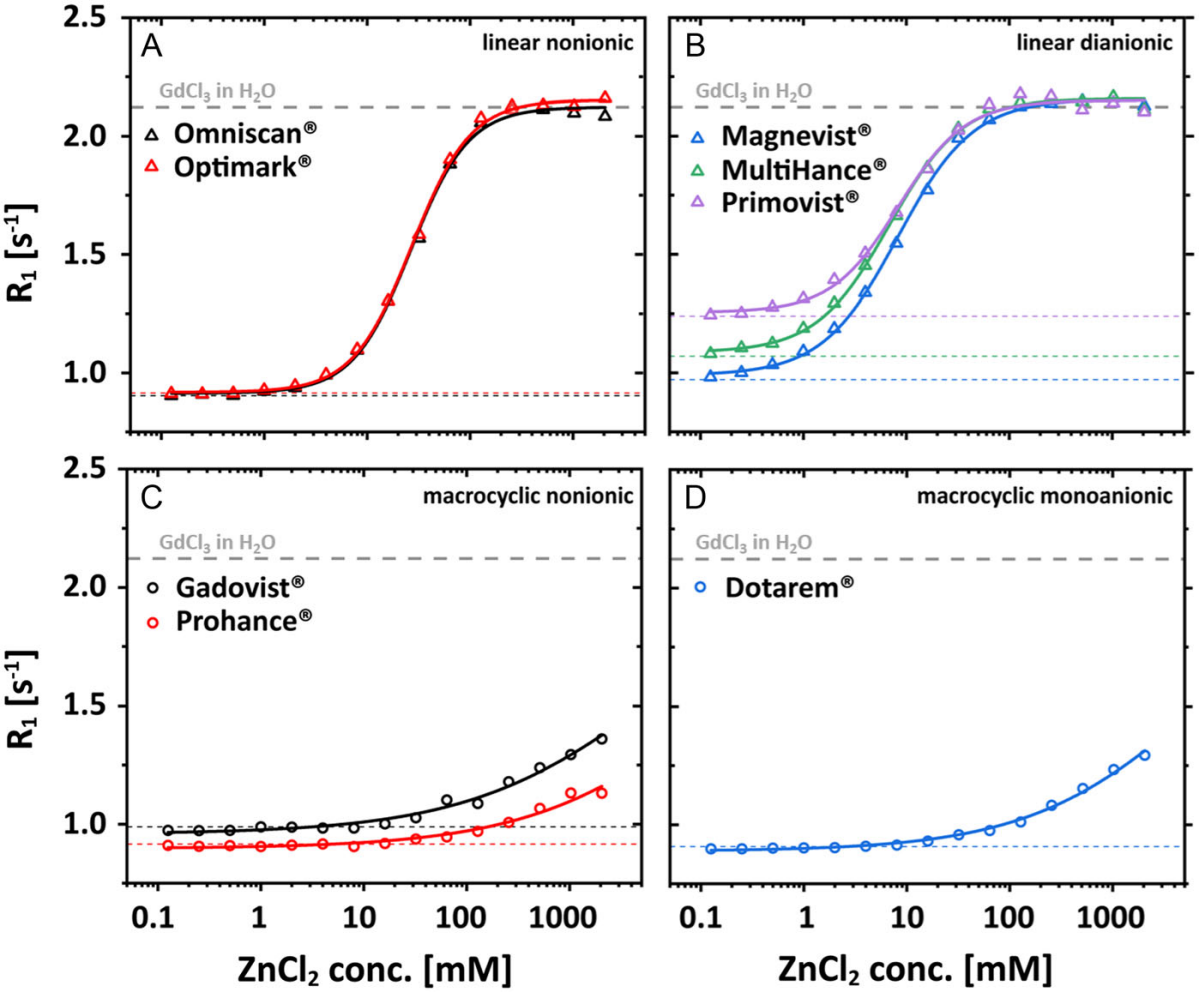
ZnCl_2_ contribution‐corrected *R*_1_ values of 150 μM GBCAs as a function of the ZnCl_2_ concentration illustrating transmetallation for (A) Omniscan (black triangle) and Optimark (red triangle); (B) Magnevist (blue triangle), MultiHance (green triangle), and Primovist (purple triangle); (C) Gadovist (black circle) and Prohance (red circle); (D) Dotarem (blue circle) in nanopure water. The dotted lines refer to the theoretical *R*_1_ values of 150 μM of the corresponding GBCA in nanopure water. The solid lines represent the fit using a logistic fit function. For Gadovist, Prohance, and Dotarem the final plateau value was fixed to the value of the theoretical *R*_1_ value of 150 μM GdCl_3_ in nanopure water (dashed gray line). The curves for Omniscan, Optimark, Magnevist, MultiHance, and Primovist reach a new plateau value of ca. *R*_1_ ≈ 2.16 ± 0.009 s^−1^ for [ZnCl_2_] > 100 mM.

Figure 3 illustrates in a systematic overview the change in *R*_1_ values as a function of ZnCl_2_ concentrations varying between 0 and 2048 mM for the different GBCAs. Linear GBCAs reach a chemical equilibrium at ZnCl_2_ concentrations >100 mM (ratio [ZnCl_2_]/[GBCA] > 670). The achieved plateau value (*R*_1_ ≈ 2.16 ± 0.009 s^−1^) for different linear GBCAs is in good agreement with the *R*_1_ value (dashed gray line) anticipated for completely dissociated Gd(III) ions (equivalent of 150 μM GdCl_3_ in nanopure water). *R*_1_ values of macrocyclic GBCAs rise significantly later than for linear GBCAs and keep increasing even at the highest administered ZnCl_2_ concentration (2048 mM, ca. 14,000‐fold excess to chelator), regardless of the GBCA's ionic status. All linear GBCAs show qualitatively the same transition in *R*_1_ for transmetallation and the curves follow very closely the course of the initially reported Magnevist behavior [19]**.** The inflection points obtained for the logistic fit function revealed that ca. 8 mM and 27 mM ZnCl_2_ are needed to induce 50% dissociation of dianionic or nonionic linear GBCAs (150 µM), respectively. This corresponds to a ~53‐ and ~180‐fold excess of ZnCl_2_ compared to the amounts of contrast agents (CA), respectively. In fact, the nonionic species appear to be more immune to the Zn(II) attack under these conditions. In comparison, macrocyclic GBCAs required extremely high concentrations of ZnCl_2_ for the transmetallation of 50% of Gd(III) ions. According to an extrapolation of the fit results, 7026 mM (46,840‐fold excess), 30,596 mM (203,973‐fold excess), and 7014 mM (46,760‐fold excess) of Zn(II) ions would be needed in the cases of Gadovist, Prohance, and Dotarem, respectively.

**FIGURE 4:**
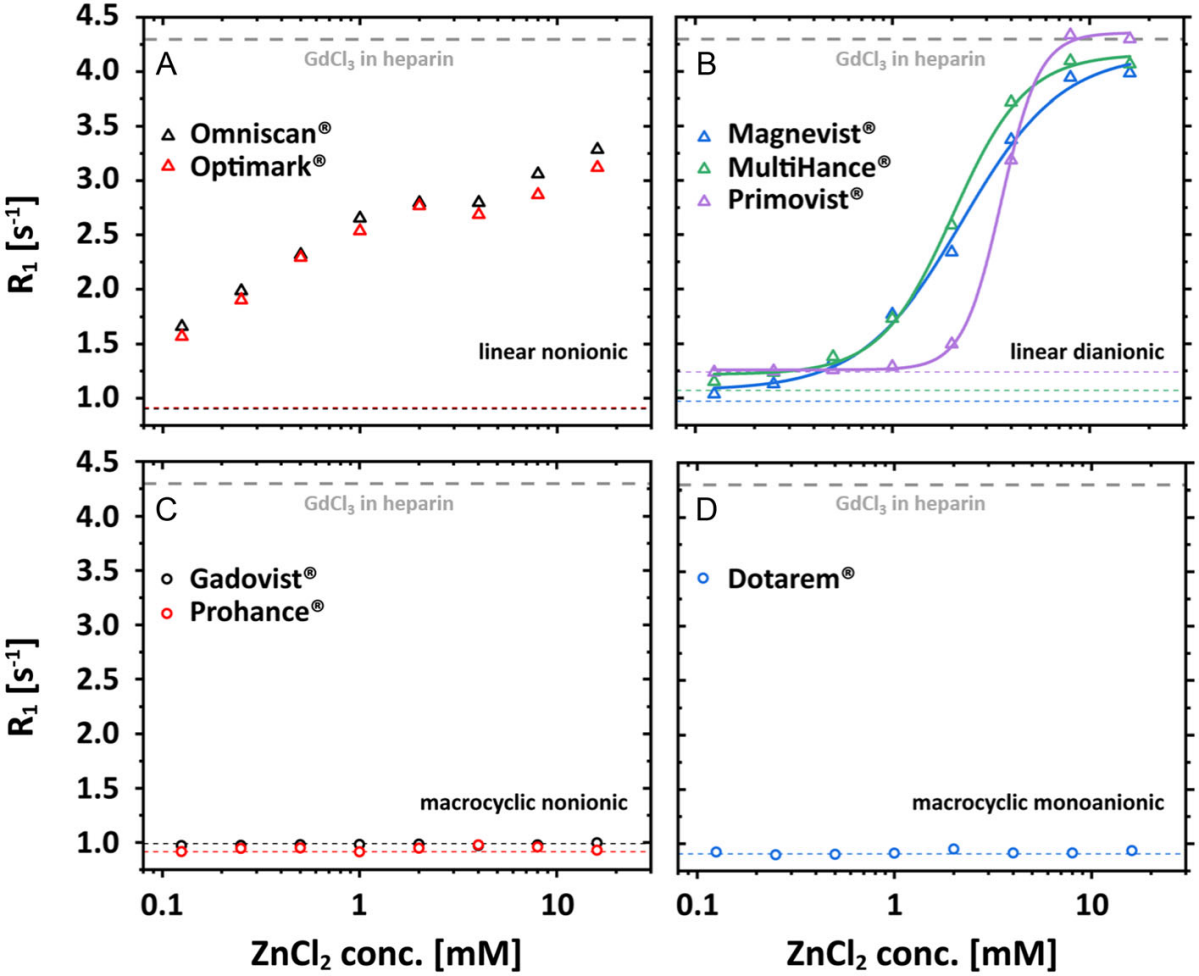
ZnCl_2_ contribution‐corrected *R*_1_ values of 150 μM GBCAs as a function of the ZnCl_2_ concentration illustrating transchelation (covering the rising shoulder in Figure 2B) for (A) Omniscan (black triangle) and Optimark (red triangle); (B) Magnevist (blue triangle), MultiHance (green triangle), and Primovist (purple triangle); (C) Gadovist (black circle) and Prohance (red circle); (D) Dotarem (blue circle) in aqueous heparin solution (100 μM). The dotted lines refer to the expected *R*_1_ values of 150 μM of the corresponding GBCA in a 100 μM heparin‐containing aqueous solution. *R*_1_ values increase and reach new plateau values at *R*_1_ ≈ 4.36 ± 0.04 s^−1^ (ZnCl_2_ > 16 mM) for Magnevist, MultiHance, and Primovist. No changes of *R*_1_ as a function of the ZnCl_2_ concentration were observed for Dotarem, Gadovist, and Prohance. Only the data points of Magnevist, MultiHance, and Primovist could be fitted using a logistic fit function represented by solid lines.

Regarding the transchelation processes, it was originally reported that the presence of the GAG reduces the thermodynamic stability and shifts the sigmoidal transition of *R*_1_ for Magnevist to smaller ZnCl_2_ concentrations [19]. This also applies when comparing Figure 2A and the rising flank in Figure 2B. While the other diionic agents Multihance and Primovist follow this trend (Figure 4B; albeit for Primovist with a later and steeper onset), we realize that contrary to the case of transmetallation, the nonionic GBCAs now do show a qualitatively different behavior: Omniscan and Optimark (Figure 4A) yield increased *R*_1_ values even for minor ZnCl_2_ concentrations, but the data do not reflect a clear transition process.

**FIGURE 5:**
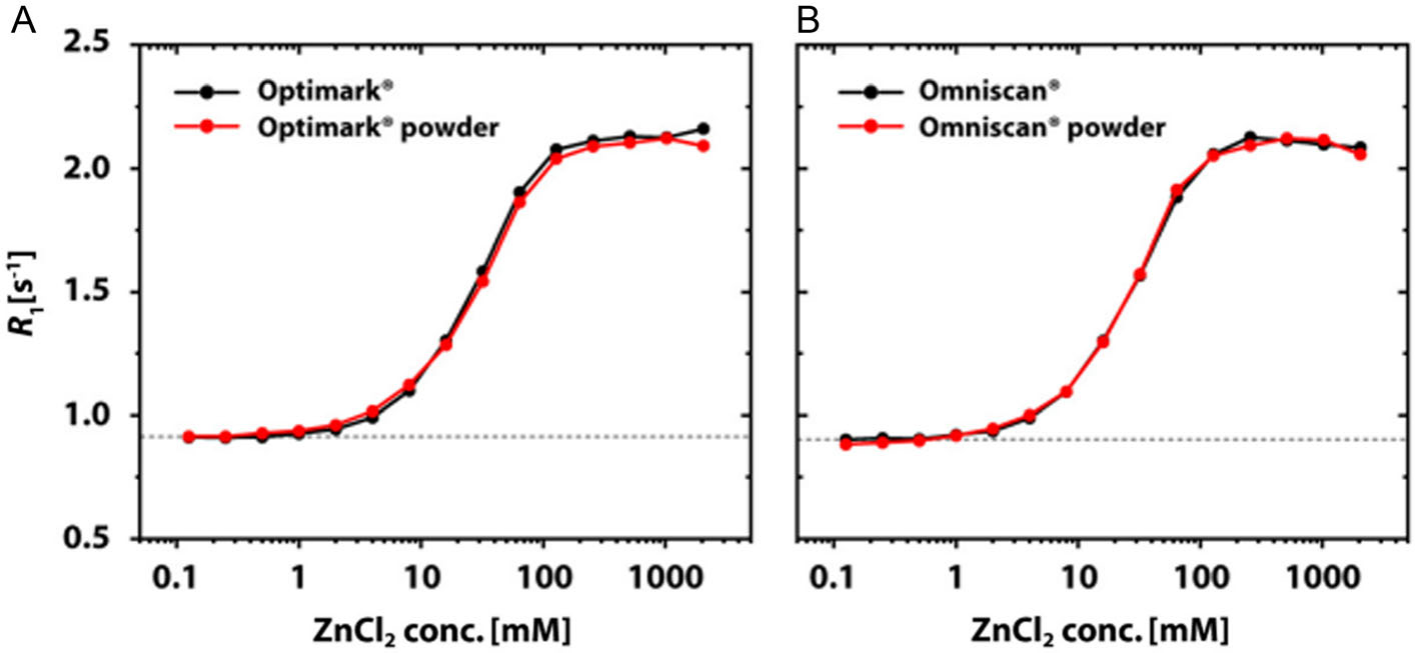
Comparison of the transmetallation processes of (A) Optimark and (B) Omniscan in nanopure water as a function of the ZnCl_2_ concentration. In (A,B), data for the clinically used formulations are shown in black and for the self‐mixed formulations with powder in red. An identical course of both curves can be observed for both GBCA.

**FIGURE 6:**
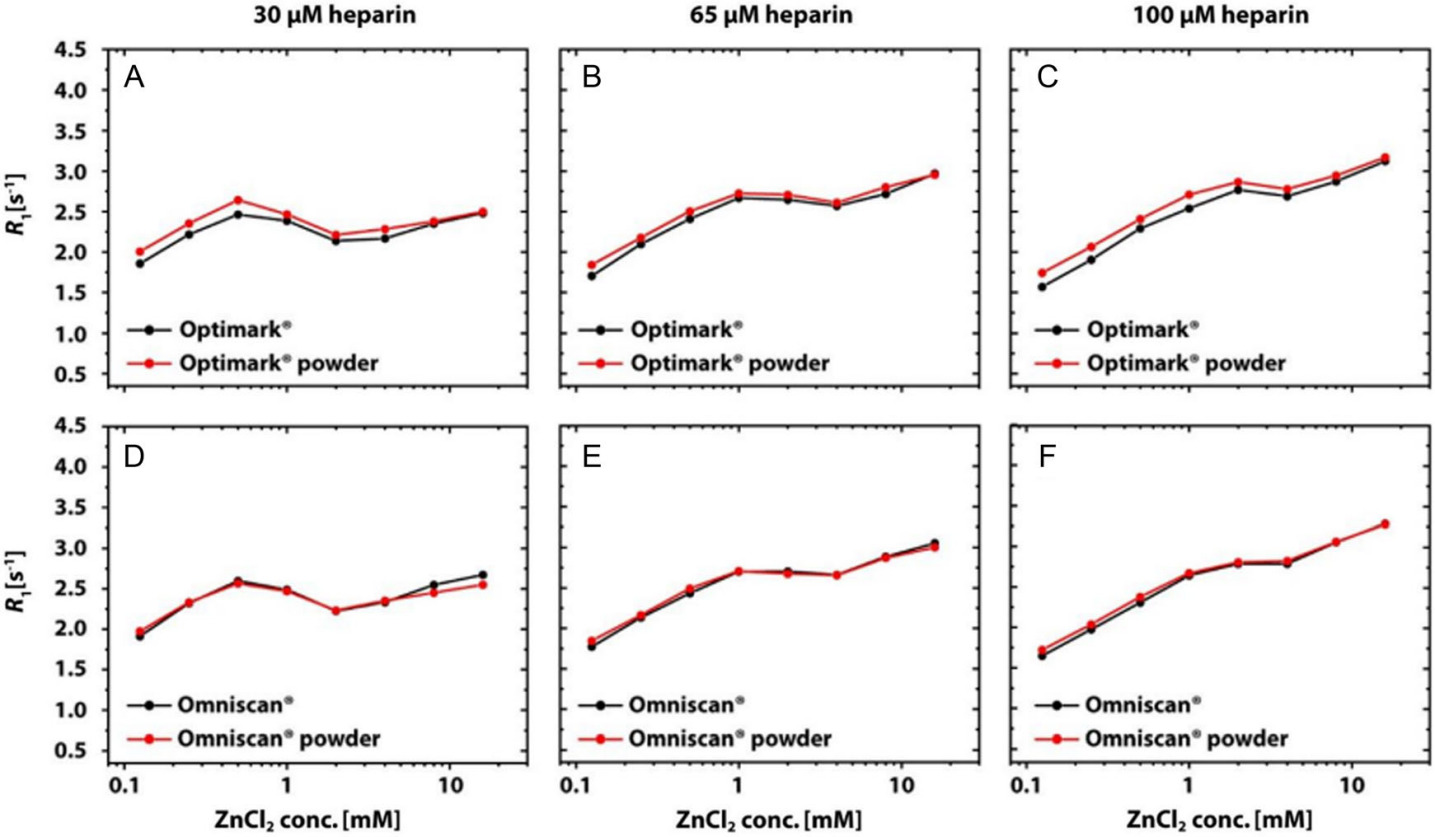
Comparison of the transchelation processes of (A–C) Optimark and (D–F) Omniscan in 30, 65, and 100 µM heparin solution as a function of the ZnCl_2_ concentration. Data for the clinically used formulations are shown in black and for the self‐mixed formulations with powder in red. An identical course of all data curves could be observed for both GBCA.

Optimark and Omniscan were therefore subjected to further investigation, as their ready‐to‐use formulations contain excess chelator [25], which may account for an altered detectability of Gd(III) ions release and heparin‐related transchelation. The corresponding transmetallation data are presented in Figure 5. The presence (in clinically used formulation) or absence (self‐mixed formulation) of excess chelator does not seem to impact the observed *R*_1_ values. The distinct transchelation behavior observed for these two GBCAs prompted further experiments using different GAG concentrations. We hypothesized that a reduced heparin concentration would make the impact of an alternative chelator more visible and compared data from three different heparin concentrations shown in Figure 6. Lower heparin concentrations are also closer to the physiologically relevant conditions. The formulation again does not have any impact. However, there are systematic changes across the different heparin concentrations, and the curves show a striking similarity in evolution across the GAG concentrations. This trend even includes an unexpected transient loss in *R*_1_ for intermediate Zn(II) concentrations for the lowest heparin content.

**FIGURE 7:**
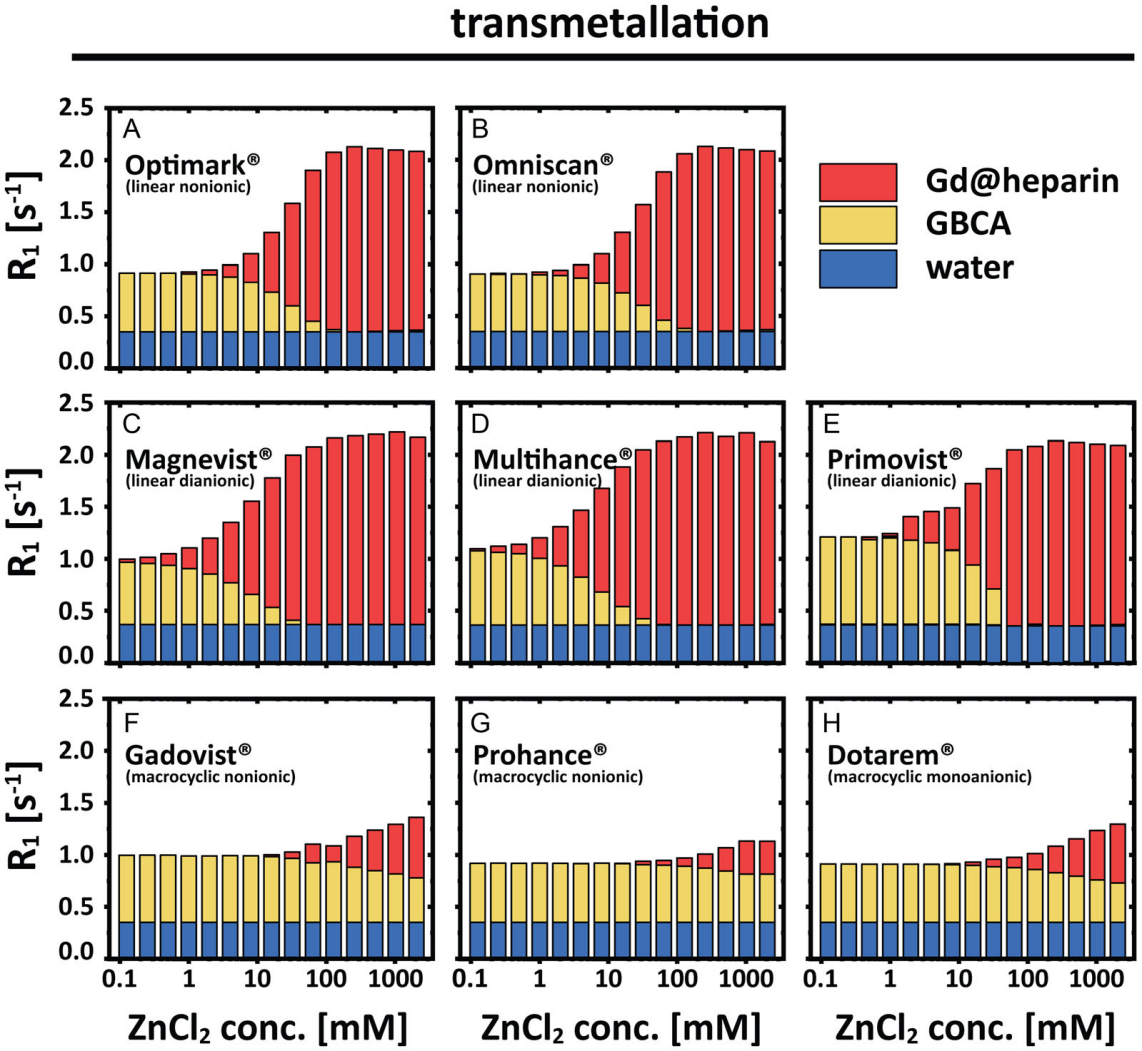
Bar charts of the individual contributions of water (blue), of the intact GBCA (yellow) and of formed Gd‐heparin complexes (red) to the overall observed relaxation rate of 150 μM (A) Optimark, (B) Omniscan, (C) Magnevist, (D) MultiHance, (E) Primovist, (F) Gadovist, (G) ProHance, and (H) Dotarem solution nanopure water.

**FIGURE 8:**
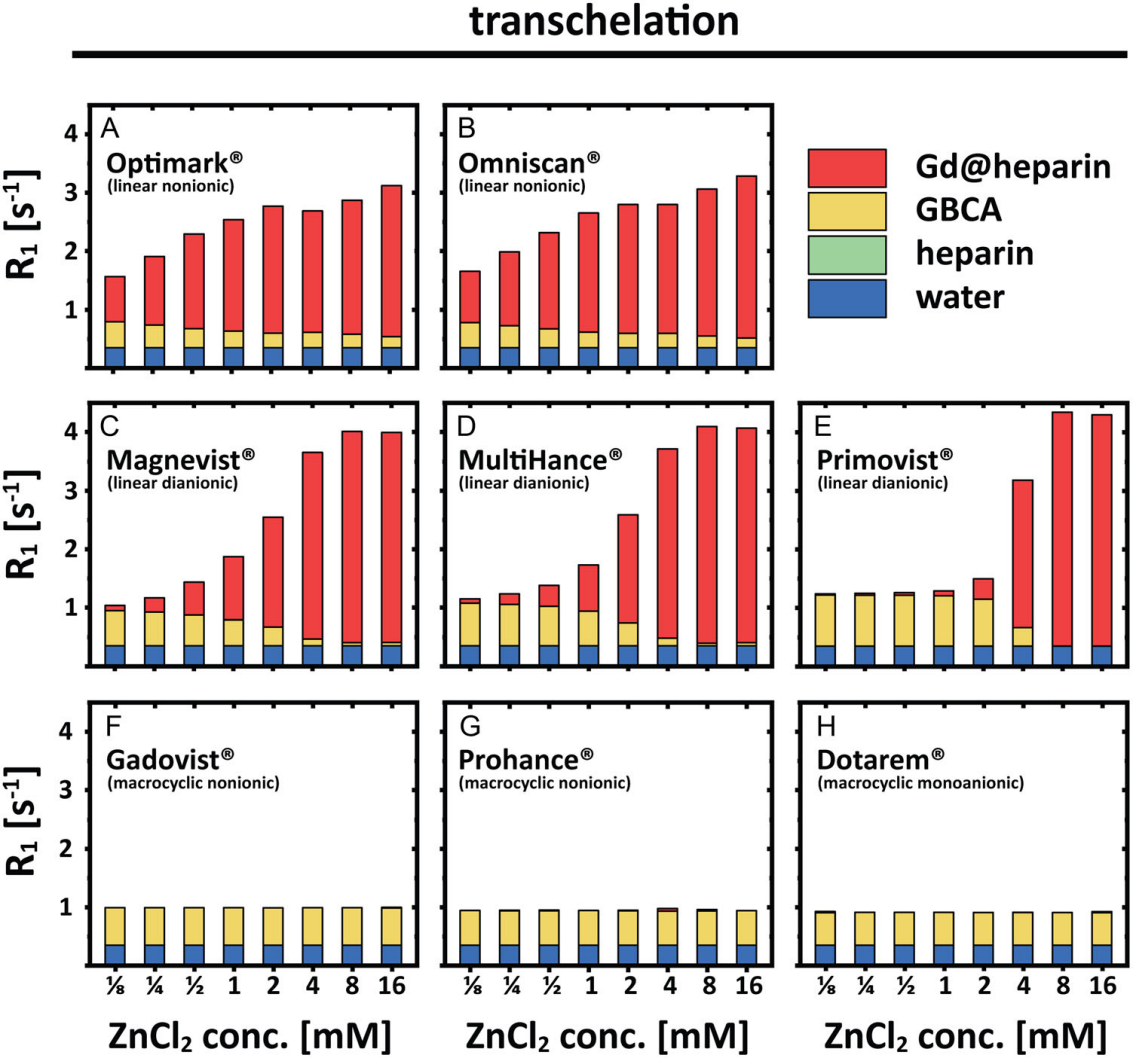
Bar charts of the individual contributions of water (blue), of heparin (green), of the intact GBCA (yellow) and of formed Gd‐heparin complexes (red) to the overall observed relaxation rate of 150 μM (A) Optimark, (B) Omniscan, (C) Magnevist, (D) MultiHance, (E) Primovist, (F) Gadovist, (G) ProHance, and (H) Dotarem solution with 100 μM heparin.

The individual *R*_1_ contributions for ZnCl_2_‐induced transmetallation of GBCAs (Figure 3) and for the overall transchelation of Gd(III) ions to heparin (Figure 4) were quantified and are illustrated as bar charts (Figures 7, 8). The contribution of pure water (blue) is independent of the utilized GBCA and the ZnCl_2_ concentration. Increasing ZnCl_2_ concentrations resulted in a decrease of the intact GBCA contributions (yellow) and an increase in the contributions of released Gd(III) ions (red). This effect is much more pronounced for linear GBCAs and starts to occur at lower ZnCl_2_ (ca. 100‐fold less) concentrations compared to macrocyclic ones. The same type of bar representation was also used for the illustration of the individual components that are involved in the overall *R*_1_ values observed after the complete transchelation process (Figure 8). Overall, systematic patterns are recognizable for nonionic versus diionic GBCAs (see discussion). In all bar charts, the contribution of heparin (*R*_1_ ≈ 0.001 s^−1^) is too small to be observable. However, it was still considered for all calculations. The bar charts illustrate that a ZnCl_2_ concentration of just 4 mM leads to an almost complete transchelation of the Gd(III) ions from linear GBCAs to heparin, while no transchelation was observed for macrocyclic GBCAs for the investigated ZnCl_2_ range.

**FIGURE 9:**
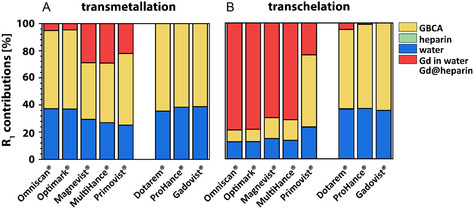
Bar charts of the actual percentage shares of the individual components of the measured relaxivity of 150 μM GBCA (yellow) solution 7 days after the addition of 2 mM ZnCl_2_ in (A) nanopure water (blue) or (B) 100 μM heparin. The percentage shares of Gd(III) ions in water and bound to heparin are represented with red bars.

**FIGURE 10:**
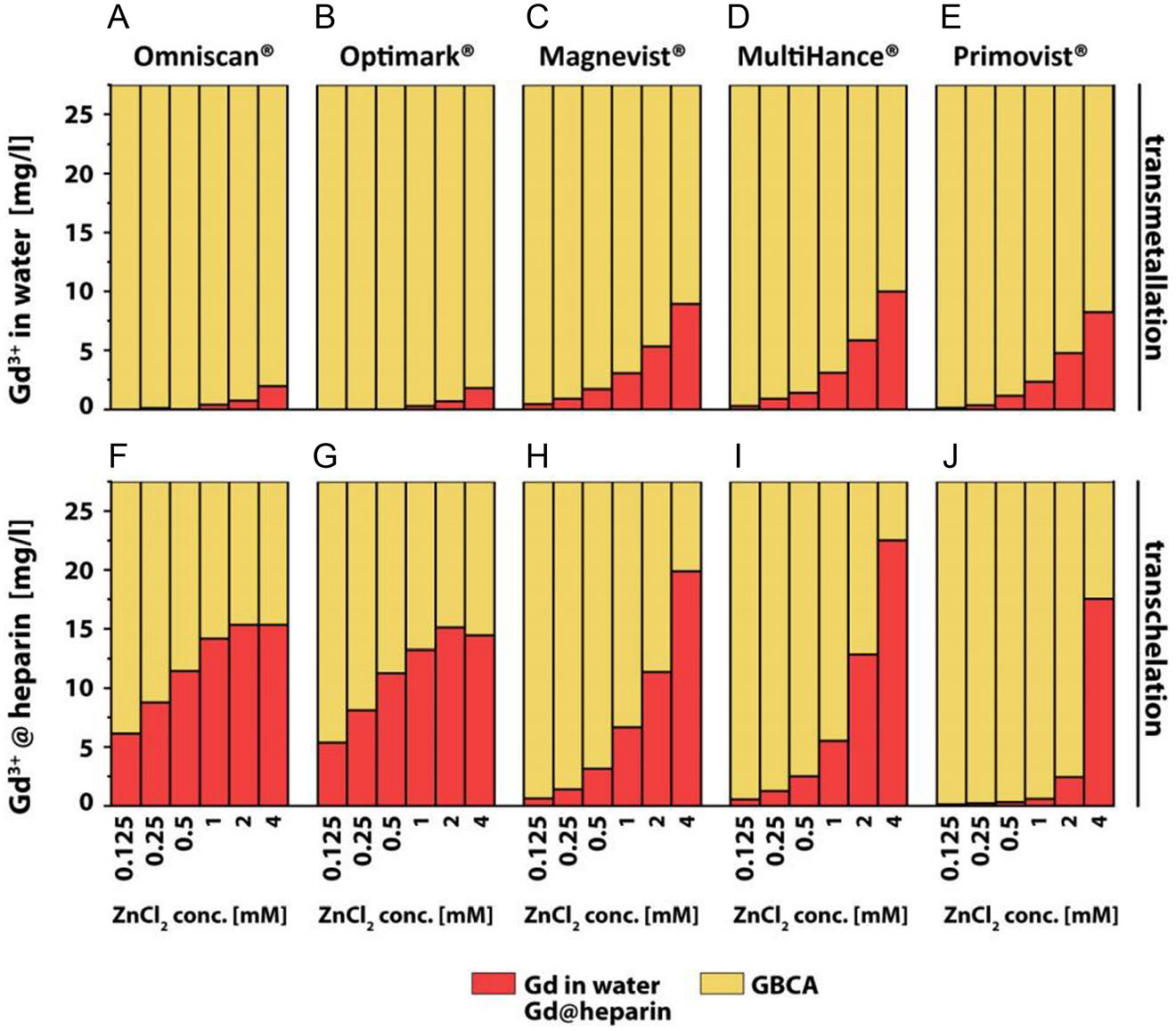
Bar charts of the actual amount (mg/l) of released or transchelated Gd(III) ions (red) from 27.9 mg/l intact (A,F) Optimark, (B,G) Omniscan, (C,H) Magnevist, (D,I) MultiHance, and (E,J) Primovist (yellow bars) after the addition of different ZnCl_2_ concentrations (0.125–4 mM). The amount of Gd(III) ions in water and bound to heparin are represented with red bars.

Complementary to the resolved absolute *R*_1_ contributions shown in Figures 7–9, the figures also show the actual percentage shares of the individual components with respect to the measured relaxivity. The results for 150 μM GBCA and 2 mM ZnCl_2_ in nanopure water (Figures 9, 10A–E) and in 100 μM heparin‐containing aqueous solution (Figure 10B–J) were selected for an exemplary visualization. The most significant differences between the transmetallation and the transchelation occurred for linear nonionic GBCAs, followed by Magnevist and MultiHance. For all macrocyclic GBCAs and Primovist, no or only minor changes were measured. As a result of the dominant *R*_1_ influence of bound Gd(III) ions in heparin, a clear decrease of the water (blue) contribution as well as a likewise declining proportion of the intact GBCA (yellow) could be observed between the different experimental setups after the addition of heparin. The relaxation contribution of intact Omniscan, Optimark, Magnevist, MultiHance, Primovist, Dotarem, Gadovist, and ProHance changes from 57%, 58%, 42%, 44%, 62%, 65%, 62%, and 62% in nanopure water to 9%, 9%, 13%, 15%, 53%, 65%, 63%, and 62% (in heparin), respectively. As a concomitant effect, the contribution of released or transchelated Gd(III) ions changes as well from 5%, 5%, 29%, 29%, 22%, 0%, 0%, 0% (Gd(III) ions in nanopure water) to 79%, 78%, 74%, 71%, 23%, 5%, 1%, 0% in heparin (Gd@heparin) containing aqueous solution, respectively. Detailed results are listed in Table S3.

### 2.3 Quantification of Dissociated Gd(III) Ions

All used equations were taken from the previously published study [19]. With reference to equations (2)–(6) and the subsequent calculation of the individual contributions of the measured relaxivity, it was possible to calculate the actual amount of released and/or transchelated Gd(III) ions. The calculated amounts in mg/l are shown in Figure 10 for the transmetallation in nanopure water (top row) and in 100 μM heparin‐containing aqueous solution (bottom row). 150 μM linear GBCA ((A,F) Omniscan, (B,G) Optimark, (C,H) Magnevist, (D,I) MultiHance, and (E,J) Primovist) and ZnCl_2_ concentrations between 0.125 and 4 mM were used for this visualization. Increasing ZnCl_2_ concentrations resulted in a decreasing amount of intact GBCA (yellow) and an increase in released/transchelated Gd(III) ions (red). These changes for Gd(III) ions are much more pronounced in experiments supplemented with heparin. The linear nonionic GBCAs show the most significant differences between the two experimental conditions: while transmetallation starts later than for the ionic GBCAs, transchelation is rather pronounced even for low Zn^2+^ concentrations. In contrast to this, the other GBCAs show only a maximum 2.3‐fold increase. The bar charts illustrate that a ZnCl_2_ concentration of 4 mM can lead to a transchelation of Gd(III) ions of up to 22 mg/l (~79%) depending on the GBCA. The amounts of Gd(III) ions released from their host structures in Omniscan, Optimark, Magnevist, MultiHance, and Primovist are ca. 2, 2, 9, 10, and 8 mg/l, respectively, in nanopure water across the set of transmetallation conditions (at highest ZnCl_2_ concentration). For the transchelation, the values range from 6, 5, 1, 1, and 0 mg/l (at 125 μM ZnCl_2_) to 16, 15, 20, 23, and 18 mg/l (at 4 mM ZnCl_2_) in heparin‐containing solutions, respectively. Thus, even 125 μM of ZnCl_2_ causes a measurable transchelation in most cases. All information given here represents rounded values; for a more detailed overview about the released or transchelated amounts of Gd(III) ions, see Table S3.

## 3 Discussion

Despite the established clinical use of GBCAs, their potential to deposit gadolinium in human tissue continues to raise important questions about long‐term safety [3, 26, 27, 28, 29]. The issue of gadolinium deposition and GDD [15] represents a complex challenge at the intersection of diagnostic innovation and patient safety. Following the introduction of a relaxometry‐based approach that demonstrated the feasibility with a diionic linear GBCA, this current study presents a comprehensive comparison of widely used GBCAs of different molecular classes and identifies groups of signatures that apparently relate to these classes.

Reliable methodologies for detecting and characterizing Gd deposition within endogenous matrices or suitable model structures are equally critical to the design of novel CAs, as they may help elucidate unresolved mechanisms. The findings presented here are based on MRI as the primary analytical technique for examining GBCA interactions with various biological substances. This approach uses the same technology that GBCAs were originally designed for, providing insights that complement and extend conventional analytical methods. A major advantage of this method is its ability to investigate multiple samples simultaneously under standardized conditions, thereby minimizing systematic bias. Furthermore, the method's nondestructive nature preserves sample integrity, allowing for repeated measurements and long‐term observational studies. As this methodology matures, its potential for use in more in vivo‐like investigations is becoming an increasingly realistic perspective. This would close an important gap, where validation of gadolinium deposition has often relied on postmortem examination in the past [30, 31].

The collected data allows quantification and comparison of the interconnected processes of transmetallation and transchelation of Gd(III) ions to GAGs for eight different GBCAs that are still in clinical use**.** The different “states”/speciations of Gd(III) ions are distinguished based on the distinct relaxivities associated with each state [19].

The relaxivity values obtained in our study show general quantitative agreement with previously reported trends in the literature [19, 20, 22] (Figure 1, Table S1). Our analysis revealed distinct differences among the individual microenvironments where water protons experience paramagnetic Gd^3+^ from different challenged GBCAs. Dissolved GdCl_3_ expectedly exhibited a significantly higher (ca. 2‐3‐fold) relaxivity (*r*_1_ ≈ 11.8 ± 0.3 s^−1^ mM^−1^) compared to GBCAs in nanopure water. This difference became even more pronounced in heparin solutions. As previously described for 100 μM heparin solutions, GdCl_3_ demonstrated a remarkable 2.5‐fold increase in relaxivity (*r*_1_ ≈ 26.3 ± 0.4 s^−1^ mM^−1^) compared to its value in water. These findings highlight the significant impact of the chemical environment on gadolinium's relaxivity, particularly the enhancing effect after heparin binding [19, 20]. These results are consistent with previous studies and support established patterns of relaxivity among the studied compounds [19, 20, 22]. However, it is important to note that direct comparisons are challenging due to variations in experimental conditions between studies. Factors such as magnetic field strength, temperature, and the medium in which the contrast agent is dissolved can significantly affect relaxivity measurements.

Nonetheless, we have been able to show that different GBCAs have different interaction profiles, reflected by *R*_1_ curves, when they face competing ions and in the presence of an alternative chelator. Our findings confirm the greater susceptibility of linear GBCAs to zinc‐induced transmetallation compared to macrocyclic GBCAs (Figures 2–8), a phenomenon observed in numerous studies**.** By utilizing ZnCl_2_ concentrations exceeding physiological levels, we achieved complete replacement of the paramagnetic ion in all linear GBCAs and substantial exchange in the macrocyclic GBCAs Gadovist, ProHance, and Dotarem (Figure 3). These three established macrocyclic GBCAs show very similar behavior and were included to illustrate the quantitative differences relative to the linear agents. The recently approved agent gadopiclenol is expected to show changes in *R*_1_ also only for rather high zinc stimuli, far beyond those required for the transmetallation or transchelation in linear GBCAs. One might consider to include gadopiclenol in future relaxivity studies for the same comparison arguments. The reliability of our study was greatly enhanced by using a wide ZnCl_2_ concentration range. This comprehensive approach allowed us to accurately extrapolate and quantify dissociated Gd(III) ions, even in cases where direct relaxivity changes were too subtle to measure, thus providing a more complete picture of gadolinium behavior under different conditions.

Initial findings for a selected linear GBCA regarding the reduced thermodynamic stability caused by the competing chelator that scavenges released Gd(III) ions can now be extended to other nonionic linear GBCAs. Notably, Primovist undergoes a rather steep transition. The behavior for nonionic linear GBCAs is, however, strikingly different but represents a common pattern for this subgroup. Figures 3A, 10A,B illustrate that in aqueous solutions (without alternative chelators), linear dianionic GBCAs (Magnevist, MultiHance, and Primovist) release higher levels of Gd(III) ions than linear nonionic GBCAs (Optimark and Omniscan) even though Optimark and Omniscan are generally considered to be highly susceptible to transmetallation reactions as linear nonionic agents. A possible explanation could have been the presence of excess chelators (not loaded with gadolinium) added by the manufacturers to the commercial formulations**.** These added chelators act as a safety mechanism to mitigate potential transmetallation reactions with endogenous competing ions, and the released Gd(III) ions should be bound again in the CA host structures instead of transchelating to other alternative chelators. Not every manufacturer uses this approach in their products. The concentrations used therefore vary approximately between 0 mol% (MultiHance), 0.2 mol% (Magnevist), 0.5 mol% (Primovist), 5 mol% (Omniscan), and 10 mol% (Optimark) [32]. To investigate the potential influence of these unoccupied chelators, we purchased the nonionic linear GBCAs in powder form without additional parent complexes. These allow experiments in addition to the clinically used formulations. Remarkably, Figures 5, 6 show that there are no discernible differences in relaxivity behavior between these samples. These results suggest that an alternative explanation, which remains unclear at present, must account for the observed phenomena. However, the signatures in Figure 6 are strikingly similar for these two GBCAs, which leads us to the idea that the underlying effects for transchelation follow a common explanation.

Another key finding of this extended study is the dual role of competing chelators, particularly GAGs. These molecules have been shown to have a remarkable ability to bind a wide range of cations [20, 33, 34, 35], making them promising candidates for binding released Gd(III) ions. As integral components of the human glycome, GAGs exert a significant influence on many biological processes. These macromolecular, highly negatively charged, endogenous polysaccharides are synthesized by all animal cells [36, 37] and have become the focus of extensive research recently [19, 20, 38, 39, 40]. GAGs are composed of repeating disaccharide subunits consisting of an amino sugar (D‐galac‐tosamine or D‐glucosamine) and a uronic acid (D‐glucuronic acid or L‐iduronic acid) linked by 1–4 glycosidic linkages [41, 42]. Within the ECM, GAGs play a pivotal role in the formation of proteoglycans, thereby contributing to the diversity and tissue distribution of the ECM [43]. As modulators of cell function, survival, and differentiation, GAGs influence overall tissue development. Their specialized functions include regulation of tumor progression, inflammation, water content, pathogen interactions, proteolysis, cell–cell recognition and signaling, and maintenance of cation homeostasis [37, 39, 44, 45, 46, 47]. Furthermore, GAGs have been shown to act as both pro‐ and anti‐inflammatory mediators [48], with elevated plasma levels observed in patients with advanced renal failure. Consequently, GAGs have been identified as potential triggers of inflammatory processes, including nephrogenic systemic fibrosis (NSF), which has been associated with Gd(III) ion deposition following administration of linear GBCAs [49]. This complex interplay highlights the multiple roles of GAGs in physiological and pathological processes, as well as their potential importance in gadolinium retention in the body. In addition to GAGs like heparin, which were studied here, other potential binding partners such as transferrin, albumin, citrate, ATP, and more should be considered and further investigated in the future as well. However, GAGs are prime candidates for binding Gd(III) ions due to their distribution, size, and chelation capacity [22, 40].

As shown previously [19], their ability to bind Gd(III) ions reduces the thermodynamic stability of the parent complex, whereas their ability to store Zn(II) ions has an opposite effect, significantly increasing kinetic stability. This Zn–heparin interaction has crucial implications for in vitro experiments and provides valuable insights. The observed increase in kinetic stability in the presence of heparin (for Magnevist, MultiHance, and Primovist) can be attributed to two possible mechanisms. Either the initial attack is inhibited by sequestration of Zn(II) ions, or the released Gd(III) ions are very slowly incorporated into the GAG matrix. But this latter explanation would only hold if bound Zn(II) ions block the Gd(III) ions from entering the GAG matrix, as shown in Figure 2B in the regime characterized by decreasing *R*_1_ that was not investigated in our previous work: The effect of additional divalent ions on the binding of Gd(III) ions to heparin becomes apparent only at concentrations above 16 mM. This observation suggests that the primary mechanism is most likely the inhibition of the initial transmetallation reaction by the sequestration of Zn(II) ions. The high concentration threshold required for Zn(II) ion interference indicates that the withholding effect is the dominant factor in modulating the reaction kinetics, rather than a direct influence on the Gd–heparin interaction.

Nevertheless, in addition, Omniscan and Optimark show a distinct “wavy” response pattern in their *R*_1_ signal, characterized by an additional plateau (Figures 4A, 6). This phenomenon, which to our knowledge has not been previously documented in the literature, suggests the presence of an additional equilibrium regime unique to these agents. It might be potentially common to the entire group of nonionic GBCAs. Our initial hypothesis regarding the influence of excess chelators on transchelation was not confirmed, and the “wave” effect (Figure 6) remains currently unexplained. In particular, these two GBCAs did not reach the relaxation rates of 150 μM Gd bound to the GAG matrix. This observation, coupled with the enhancement of the “wave” effect at lower heparin concentrations, suggests the possibility of additional interactions or intermediates that hinder the interaction with the GAG as an alternative chelator. The limited scavenging capacity of 35 µM heparin exhibits the most pronounced transient dip in *R*_1_. Increasing exposure to Zn(II) at the GAG binding sites could lead to blockage for Gd binding and also to cross‐linking of GAG units. The latter is expected to reduce tumbling and thus even increase *R*_1_, which does not match the observation of a transient loss or stagnation in *R*_1_. The former could represent the beginning of what also causes the relapse of *R*_1_ in Figure 2B for [Zn^2+^] > 16 µM. However, it remains unclear why this effect is of such transient nature, and further addition of Zn^2+^ eventually yields increasing *R*_1_. Further investigation using additional analytical methods is planned to elucidate the chemical interactions underlying these effects.

Future studies should broaden the scope of investigation to include additional physiologically relevant cations such as Cu^2+^, Ca^2+^, Fe^3+^, and Mg^2+^, which may also compete with GBCAs and thereby influence the extent of transmetallation and transchelation. Moreover, extending the experimental design to encompass a broader range of GAG concentrations, as well as structurally diverse GAG species, could help to delineate the role of macromolecular diversity in gadolinium binding and deposition. In a further step, the integration of more complex model systems, such as hydrogels or other biomimetic matrices, may provide a closer approximation to physiological environments. Together, these efforts will be instrumental in refining our understanding of the molecular fate of GBCAs and in identifying potential risks and stabilization strategies with higher translational relevance.

## 4 Conclusion

The issue of gadolinium deposition represents a complex challenge at the intersection of diagnostic innovation and patient safety. The ongoing discussion concerning GDD and symptoms associated with gadolinium exposure (SAGE [50]) underlines the need for further investigations that cover mechanistic understanding in simplified model systems and reach toward more complex conditions in vivo.

The present study complements findings published earlier for only two selected GBCAs [19] and reveals previously undescribed patterns in systematic water relaxometry experiments that sense the transition between different chemical equilibria based on the different relaxivities of Gd(III) ions in various molecular environments induced by ZnCl_2_. The relaxometry‐based approach is a powerful tool with great potential for future research in this field. It can be expanded to every new GBCA and even for more complex experimental environments. It facilitates the quantification of equilibrium concentrations and kinetics associated with three pivotal processes: ion‐induced transmetallation of GBCAs, binding of Gd(III) ions to macromolecular structures such as GAGs, and the combination as a transchelation process from GBCAs to macromolecules. This comprehensive analysis provides a more nuanced picture of GBCA behavior than the previous one based on just two agents that did not cover all four classes. Furthermore, this method can help to facilitate the identification of effective competing ions and potent Gd‐complexing macromolecules, in addition to the assessment of their stabilizing and destabilizing effects on GBCAs. It also demonstrates that complete transmetallation of GBCAs may be associated with reduced *R*_1_ values when Gd transchelation is impaired. With this study, we could show a first step with an easy to implement and versatile experimental design in order to gain fundamental insights. In the future, a stepwise increase in experimental complexity is planned to gradually bridge the gap between controlled laboratory conditions and real physiological environments. The aim is to develop a robust and adaptable experimental protocol that will provide increasingly comprehensive insights into the complex biochemical mechanisms. Future iterations of the study will focus on integrating more sophisticated experimental parameters, potentially including multilevel molecular interactions, more diverse chelator systems and more accurately simulated biological conditions.

By exploring these interactions between endogenous substances and administered CA, we are able to add and illuminate another aspect in the field of GBCA safety which was not addressed in this way in the past. This information can be important in the development of new, more stable GBCAs, as well as in refining our understanding of the potential risks associated with existing agents.

## 5 Experimental Section

5.1

The experimental setup, conditions, and equations used in this study were identical to those described earlier [19]. Therefore, only essential information is repeated or added in this study, including the materials and equipment used and the pulse sequence parameters. Detailed information on the implementation of the following individual experiments can be found in the previous publication. This contains transmetallation experiments, individual relaxation determinations, quantification of the released Gd(III) ions, transchelation experiments, and quantification of the transchelated Gd(III) ions.

### 5.2 Materials

All used CA in this study were obtained as solutions for medical injections from their respective pharmaceutical distributors: Gd‐DOTA (Dotarem, 0.5 mol L^−1^) from Guerbet GmbH (Sulzbach, Germany), Gd‐BOPTA (MultiHance, 0.5 mol L^−1^), and Gd‐HP‐DO3A (ProHance, 0.5 mol L^−1^) from Bracco Imaging (Konstanz, Germany), Gd‐DTPA‐BMA (Omniscan, 0.5 mol L^−1^) from GE Healthcare Buchler GmbH & Co. KG (Brausnschweig, Germany), Gd‐DTPA‐BMEA (Optimark 0.5 mol L^−1^) from Mallinckrodt Deutschland GmbH (Hennef, Germany), Gd‐BT‐DO3A (Gadovist, 1.0 mol L^−1^), Gd‐DTPA (Magnevist, 0.5 mol L^−1^) and Gd‐EOB‐DTPA (Primovist, 0.25 mol L^−1^) from Bayer Vital GmbH (Leverkusen, Germany). To investigate a potential effect of empty chelators, in addition to the clinically used formulations, the nonionic linear GBCAs were also purchased as powder without additional GBCA parent complexes. The trade names of all GBCAs are used throughout this study, as all chemicals were used as received without any further purification or modification of their chemical structures. GdCl_3_ (gadolinium(III)chloride hexahydrate, 99% titration) and ZnCl_2_ (zinc chloride puriss.) served as sources for free ions (both salts purchased from Sigma‐Aldrich Chemie GmbH, Steinheim, Germany). As example for a human endogenous GAG, a commercially available heparin solution (Heparin‐Natrium‐250,000‐ratiopharm, 250,000 IU/mL, average molecular weight MW = 13 kDa, Ratiopharm GmbH, Ulm, Germany) was used. Nanopure water (18 MΩ cm) was used for the preparation of all model solutions.

### 5.3 MRI Relaxometry Mapping

All measurements of the water proton *T*_1_ relaxation time were conducted using a 25 mm double‐resonant ^1^H/^129^Xe coil (^129^Xe channel not used) with a dephasing‐recovery pulse sequence that included 50 π/2 pulses, followed by gradient spoiling and a gradient echo (GRE)‐based, centric‐reordered image readout. The parameters for the image readout were as follows: field of view (FoV) of 20 × 20 mm^2^, matrix size of 128 x 128, slice thickness of 2 mm, bandwidth (BW) of 50 kHz, echo time (TE) of 2.5 ms, and repetition time (TR) of 5.7 ms. A variable temperature unit (VTU) was utilized to regulate the temperature of the sample. Unless otherwise indicated, measurements were conducted approximately 1 h after the sample solutions were placed in the magnet to ensure a stable temperature of 25°C. Quantitative *R*_1_ values (where *R*_1_ = 1/*T*_1_) were calculated by fitting a monoexponential function to the region of interest (ROI)‐averaged data collected at various inversion times ranging from 10 ms to 6 s. The presented values are the mean (±1 standard deviation) calculated from 10 independently obtained *T*_1_ maps.

## Supporting Information

Additional supporting information can be found online in the Supporting Information section. **Supporting** **Fig.** **S1**: Representation of the influence of ZnCl_2_ (0.125–2046 mM) on the pH values in solutions with Dotarem, Magnevist, Gadovist and Optimark in (A) milli‐Q water and (B). 100 μM heparin solution. Decreasing pH values can be observed with increasing ZnCl_2_ concentrations. **Supporting** **Table** **S1**: Rounded r_1_ values of all used GBCAs in nanopure water as well as in heparin. **Supporting** **Table** **S2**: Determination of the amount in mg/l of intact GBCA and the dissociated Gd(III)‐ions in the presence of various ZnCl without (transmetallation) constant heparin (100 mM) concentrations. **Supporting** **Table** **S3**: Table of the percentage contributions during the transmetallation in nano pure water as well as the transchelation in 100 µM heparin solution and constant 2 mM ZnCl_2_ concentration.

## Funding

This project was partially funded by the Deutsche Forschungsgemeinschaft (DFG, German Research Foundation) through grant nos. 289347353 (GRK 2260) and 372486779 (SFB 1340) and also received support by the Dieter Morszeck Stiftung.

## Conflicts of Interest

The author declares no conflicts of interest.

## Supporting information

Supplementary Material

## Data Availability

The data that support the findings of this study are available from the corresponding author upon reasonable request.

## References

[1] M. F. Bellin , “MR Contrast Agents, the Old and the New,” European Journal of Radiology 60 (2006): 314–323. 17005349 10.1016/j.ejrad.2006.06.021

[2] L. M. De Leõn‐Rodríguez , A. F. Martins , M. C. Pinho , N. M. Rofsky , and A. D. Sherry , “Basic MR Relaxation Mechanisms and Contrast Agent Design,” Journal of Magnetic Resonance Imaging 42 (2015): 545–565. 25975847 10.1002/jmri.24787 PMC4537356

[3] J. Ramalho , R. C. Semelka , M. Ramalho , R. H. Nunes , M. AlObaidy , and M. Castillo , “Gadolinium‐Based Contrast Agent Accumulation and Toxicity: An Update,” American Journal of Neuroradiology 37 (2016): 1192–1198. 26659341 10.3174/ajnr.A4615 PMC7960350

[4] M. Rogosnitzky and S. Branch , “Gadolinium‐Based Contrast Agent Toxicity: A Review of Known and Proposed Mechanisms,” Biometals 29 (2016): 365–376. 27053146 10.1007/s10534-016-9931-7 PMC4879157

[5] M. F. Bellin and A. J. Van Der Molen , “Extracellular Gadolinium‐Based Contrast Media: An Overview,” European Journal of Radiology 66 (2008): 160–167. 18358659 10.1016/j.ejrad.2008.01.023

[6] F. G. Shellock and E. Kanal , “Safety of Magnetic Resonance Imaging Contrast Agents,” Journal of Magnetic Resonance Imaging 10 (1999): 477–484. 10508312 10.1002/(sici)1522-2586(199909)10:3<477::aid-jmri33>3.0.co;2-e

[7] H. S. Thomsen , “Are the Increasing Amounts of Gadolinium in Surface and Tap Water Dangerous?,” Acta Radiologica 58 (2017): 259–263. 27609906 10.1177/0284185116666419

[8] J. M. Idée , M. Port , C. Medina , et al., “Possible Involvement of Gadolinium Chelates in the Pathophysiology of Nephrogenic Systemic Fibrosis: A Critical Review,” Toxicology 248 (2008): 77–88. 18440117 10.1016/j.tox.2008.03.012

[9] R. Brünjes and T. Hofmann , “Anthropogenic Gadolinium in Freshwater and Drinking Water Systems,” Water Research 182 (2020): 115966. 32599421 10.1016/j.watres.2020.115966 PMC7256513

[10] J. Wahsner , E. M. Gale , A. Rodríguez‐Rodríguez , and P. Caravan , “Chemistry of MRI Contrast Agents: Current Challenges and New Frontiers,” Chemical Reviews 119 (2019): 957–1057. 30350585 10.1021/acs.chemrev.8b00363 PMC6516866

[11] C. A. Mallio , P. M. Parizel , C. C. Quattrocchi , and B. G. Deposition , “Gadolinium Deposition Safety : Seeking the Patient's Perspective,” American Journal of Neuroradiology 41 (2020): 27–29. 10.3174/ajnr.A6586 PMC7342758 32381539

[12] A. N. Oksendal and P.‐A. Hals , “Biodistribution and Toxicity of MR Imaging Contrast Media,” Journal of Magnetic Resonance Imaging 3 (1993): 157–165. 8428083 10.1002/jmri.1880030128

[13] K. S. Park , Y. J. Heo , H. W. Jeong , J. W. Baek , H. J. Choo , and C. S. Jung , “Retention of Gadolinium in Cerebrospinal Fluid and Decreased Renal Function: A Case Report,” Journal of the Korean Society of Radiology 77 (2017): 348–348.

[14] R. C. Semelka , M. Ramalho , and M. Jay , “Summary of Special Issue on Gadolinium Bioeffects and Toxicity with a Look to the Future,” Magnetic Resonance Imaging 34 (2016): 1399–1401. 27639920 10.1016/j.mri.2016.09.002

[15] R. C. Semelka , J. Ramalho , A. Vakharia , et al., “Gadolinium Deposition Disease: Initial Description of a Disease that Has Been around for a while,” Magnetic Resonance Imaging 34 (2016): 1383–1390. 27530966 10.1016/j.mri.2016.07.016

[16] A. D. Sherry , P. Caravan , and R. E. Lenkinski , “Primer on Gadolinium Chemistry,” Journal of Magnetic Resonance Imaging 30 (2009): 1240–1248. 19938036 10.1002/jmri.21966 PMC2853020

[17] S. Laurent , L. V. Elst , C. Henoumont , and R. N. Muller , “How to Measure the Transmetallation of a Gadolinium Complex,” Contrast Media & Molecular Imaging 5 (2010): 305–308, 10.1002/cmmi.388. 20803503

[18] S. Laurent , L. V. Elst , F. Copoix , and R. N. Muller , “Stability of MRI Paramagnetic Contrast Media: A Proton Relaxometric Protocol for Transmetallation Assessment,” Investigative Radiology 36 (2001): 115–122. 11224760 10.1097/00004424-200102000-00008

[19] P. Werner , M. Taupitz , L. Schröder , and P. Schuenke , “An NMR Relaxometry Approach for Quantitative Investigation of the Transchelation of Gadolinium Ions from GBCAs to a Competing Macromolecular Chelator,” Scientific Reports 11 (2021): 21731. 34741037 10.1038/s41598-021-00974-4 PMC8571392

[20] M. Taupitz , N. Stolzenburg , M. Ebert , et al., “Gadolinium‐Containing Magnetic Resonance Contrast Media: Investigation on the Possible Transchelation of Gd3+ to the Glycosaminoglycan Heparin,” Contrast Media & Molecular Imaging 8 (2013): 108–116. 23281283 10.1002/cmmi.1500

[21] M. Le Fur and P. Caravan , “The Biological Fate of Gadolinium‐Based MRI Contrast Agents: A Call to Action for Bioinorganic Chemists,” Metallomics 11 (2019): 240–254. 30516229 10.1039/c8mt00302e PMC6486840

[22] P. Werner , P. Schuenke , O. Krylova , H. Nikolenko , M. Taupitz , and L. Schröder , “Investigating the Role of Sulfate Groups for the Binding of Gd3+ Ions to Glycosaminoglycans with NMR Relaxometry,” ChemMedChem 17 (2022): e202100764, 10.1002/cmdc.202100764. 35451227 PMC9400987

[23] J. Garcia , S. Z. Liu , and A. Y. Louie , “Biological Effects of MRI Contrast Agents: Gadolinium Retention, Potential Mechanisms and a Role for Phosphorus,” Philosophical Transactions. Series A, Mathematical, Physical, and Engineering Sciences 375 (2017): 375, 10.1098/rsta.2017.0180. PMC5647271 29038383

[24] G. W. White , W. A. Gibby , and M. F. Tweedle , “Comparison of Gd(DTPA‐BMA) (Omniscan) versus Gd(HP‐DO3A) (ProHance) Relative to Gadolinium Retention in Human Bone Tissue by Inductively Coupled Plasma Mass Spectroscopy,” Investigative Radiology 41 (2006): 272–278. 16481910 10.1097/01.rli.0000186569.32408.95

[25] S. K. Morcos , “Nephrogenic Systemic Fibrosis following the Administration of Extracellular Gadolinium Based Contrast Agents: Is the Stability of the Contrast Agent Molecule an Important Factor in the Pathogenesis of This Condition?,” The British Journal of Radiology 80 (2007): 73–76. 17392401 10.1259/bjr/17111243

[26] T. Kanda , K. Ishii , H. Kawaguchi , K. Kitajima , and D. Takenaka , “High Signal Intensity in the Dentate Nucleus and Globus Pallidus on Unenhanced T1‐Weighted MR Images: Relationship with Increasing Cumulative Dose of a Gadoliniumbased Contrast Material,” Radiology 270 (2014): 834–841. 24475844 10.1148/radiol.13131669

[27] T. Sato , K. Ito , T. Tamada , et al., “Tissue Gadolinium Deposition in Renally Impaired Rats Exposed to Different Gadolinium‐Based MRI Contrast Agents: Evaluation with Inductively Coupled Plasma Mass Spectrometry (ICP‐MS,” Magnetic Resonance Imaging 31 (2013): 1412–1417. 23643157 10.1016/j.mri.2013.03.025

[28] C. Thakral and J. L. Abraham , “Automated Scanning Electron Microscopy and X‐Ray Microanalysis for In Situ Quantification of Gadolinium Deposits in Skin,” Journal of Electron Microscopy 56 (2007): 181–187. 17951398 10.1093/jmicro/dfm020

[29] S. Sanyal , P. Marckmann , S. Scherer , and J. L. Abraham , “Multiorgan Gadolinium (Gd) Deposition and Fibrosis in a Patient with Nephrogenic Systemic Fibrosis ‐ An Autopsy‐Based Review,” Nephrology, Dialysis, Transplantation 26 (2011): 3616–3626. 10.1093/ndt/gfr085 21441397

[30] R. J. McDonald , J. S. McDonald , D. F. Kallmes , et al., “Intracranial Gadolinium Deposition after Contrast‐Enhanced MR Imaging,” Radiology 275 (2015): 772–782. 25742194 10.1148/radiol.15150025

[31] T. Kanda , M. Matsuda , H. Oba , K. Toyoda , and S. Furui , “Gadolinium Deposition after Contrastenhanced MR Imaging,” Radiology 277 (2015): 924–925. 26599932 10.1148/radiol.2015150697

[32] Q. N. Do , R. E. Lenkinski , G. Tircso , and Z. Kovacs , “How the Chemical Properties of GBCAs Influence Their Safety Profiles In Vivo,” Molecules 27 (2022): 58. 10.3390/molecules27010058 PMC8746842 35011290

[33] P. T. Doganes and M. Schubert , “The Use of Lanthanum to Study the Degradation of a Proteinpolysaccharide from Cartilage,” The Journal of Biological Chemistry 239 (1964): 1498–1503. 14189883

[34] R. N. Rej , K. R. Holme , and A. S. Perlin , “Marked Stereoselectivity in the Binding of Copper Ions by Heparin. Contrasts with the Binding of Gadolinium and Calcium Ions,” Carbohydrate Research 207 (1990): 143–152. 2076515 10.1016/0008-6215(90)84044-u

[35] D. L. Rabenstein , J. M. Robert , and J. Peng , “Multinuclear Magnetic Resonance Studies of the Interaction of Inorganic Cations with Heparin,” Carbohydrate Research 278 (1995): 239–256. 8590444 10.1016/0008-6215(95)00263-4

[36] A. D. Lander and S. B. Selleck , “The Elusive Functions of Proteoglycans: In Vivo Veritas,” Journal of Cell Biology 148 (2000): 227–232. 10648554 10.1083/jcb.148.2.227 PMC2174291

[37] L. Zhang , Glycosaminoglycan (GAG) Biosynthesis and GAG‐Binding Proteins. Elsevier Inc, 2010). 10.1016/S1877-1173(10)93001-9 20807638

[38] M. Lima , T. Rudd , and E. Yates , “New Applications of Heparin and Other Glycosaminoglycans,” Molecules 22 (2017): 1–11. 10.3390/molecules22050749 PMC6154012 28481236

[39] D. Soares da Costa , R. L. Reis , and I. Pashkuleva , “Sulfation of Glycosaminoglycans and Its Implications in Human Health and Disorders,” Annual Review of Biomedical Engineering 19 (2017): 1–26. 10.1146/annurev-bioeng-071516-044610 28226217

[40] L. Polewski , D. Dymnikova , W. Malicka , et al., Analytical Chemistry 97 (2025): 11436–11442. 40446135 10.1021/acs.analchem.4c06624 PMC12163887

[41] M. Mende , C. Bednarek , M. Wawryszyn , et al., “Chemical Synthesis of Glycosaminoglycans,” Chemical Reviews 116 (2016): 8193–8255. 27410264 10.1021/acs.chemrev.6b00010

[42] V. H. Pomin and B. Mulloy , “Glycosaminoglycans and Proteoglycans,” Pharmaceuticals 11 (2018): 1–9. 10.3390/ph11010027 PMC5874723 29495527

[43] N. S. Gandhi and R. L. Mancera , “The Structure of Glycosaminoglycans and Their Interactions with Proteins,” Chemical Biology & Drug Design 72 (2008): 455–482. 19090915 10.1111/j.1747-0285.2008.00741.x

[44] R. S. Aquino , E. S. Lee , and P. W. Park , Diverse Functions of Glycosaminoglycans in Infectious Diseases. Elsevier Inc, 2010). 10.1016/S1877-1173(10)93016-0 20807653

[45] R. Vives , H. Lortat‐Jacob , and P. Fender , “Heparan Sulphate Proteoglycans and Viral Vectors : Ally or Foe?,” Current Gene Therapy 6 (2006): 35–44. 16475944 10.2174/156652306775515565

[46] D. Spillmann , “Heparan Sulfate: Anchor for Viral Intruders?,” Biochimie 83 (2001): 811–817. 11530214 10.1016/s0300-9084(01)01290-1

[47] R. V. Iozzo , “Basement Membrane Proteoglycans: From Cellar to Ceiling,” Nature Reviews Molecular Cell Biology 6 (2005): 646–656. 16064139 10.1038/nrm1702

[48] K. R. Taylor and R. L. Gallo , “Glycosaminoglycans and Their Proteoglycans: Host‐Associated Molecular Patterns for Initiation and Modulation of Inflammation,” FASEB Journal 20 (2006): 9–22. 16394262 10.1096/fj.05-4682rev

[49] J.‐M. Idée , N. Fretellier , C. Robic , and C. Corot , “The Role of Gadolinium Chelates in the Mechanism of Nephrogenic Systemic Fibrosis: A Critical Update,” Critical Reviews in Toxicology 44 (2014): 895–913. 25257840 10.3109/10408444.2014.955568

[50] R. J. McDonald , J. C. Weinreb , and M. S. Davenport , “Symptoms Associated with Gadolinium Exposure (SAGE): A Suggested Term,” Radiology 302 (2022): 270–273. 34783590 10.1148/radiol.2021211349

