# Supplementary material for: Comprehensive Magnetic Resonance Imaging Relaxometry of Gadolinium‐Based Contrast Agents: A Systematic Study of Transmetallation and Transchelation Processes With Zinc Ions and Heparin
Source: ChemMedChem. 2026 Mar 26;21(6):e202501096. doi: 10.1002/cmdc.202501096 (PMC13020528; doi:10.1002/cmdc.202501096)
Supplement: Supplementary file 1 — Supplementary Material [file CMDC-21-e202501096-s001.pdf]

# Supporting Information

for

# Comprehensive MRI Relaxometry of Gadolinium-Based Contrast Agents: A Systematic Study of Transmetallation and Transchelation Processes with Zinc Ions and Heparin

Patrick Werner,[b] and Leif Schröder*[a]

[a] Prof. Dr. rer. nat. L. Schröder
Translational Molecular Imaging
Deutsches Krebsforschungszentrum
Im Neuenheimer Feld 280, 69120 Heidelberg, Germany

Department of Physics and Astronomy
Ruprecht-Karls Universität Heidelberg
Heidelberg, Germany

German Cancer Consortium (DKTK) Partner Site Heidelberg,
Heidelberg, Germany
E-mail: leif.schroeder@dkfz.de

[b] Dr. rer. nat. P. Werner
Translational Molecular Imaging
Deutsches Krebsforschungszentrum
Im Neuenheimer Feld 280, 69120 Heidelberg, Germany

Department of Radiology, Charité – Universitätsmedizin Berlin,
Luisenstr. 5, 10117 Berlin, Germany

**SuppTable 1:** Rounded $r_1$ values of all used GBCAs in nanopure water as well as in heparin.

| Gadolinium based Contrast Agents (GBCAs) | | $H_2O$ | | | heparin (100 µM) | | |
|---|---|---|---|---|---|---|---|
| Trademarks | Acronyms | $r_1$ [$s^{-1}$ $mM^{-1}$] | ± | $R^2$ | $r_1$ [$s^{-1}$ $mM^{-1}$] | ± | $R^2$ |
| Omniscan® | Gd-DTPA-BMA | 3.7 | 0.3 | 0.999 | 3.7 | 0.3 | 0.999 |
| Optimark® | Gd-DTPA-BMEA | 3.8 | 0.3 | 0.999 | 3.7 | 0.4 | 0.999 |
| MultiHance® | Gd-BOPTA | 4.8 | 0.3 | 0.999 | 4.9 | 0.3 | 0.999 |
| Magnevist® | Gd-DTPA | 4.1 | 0.3 | 0.999 | 4.1 | 0.3 | 0.992 |
| Primovist® | Gd-EOB-DTPA | 5.9 | 0.3 | 0.999 | 5.8 | 0.3 | 0.999 |
| Gadovist® | Gd-BT-DO3A | 4.3 | 0.3 | 0.999 | 4.3 | 0.4 | 0.999 |
| ProHance® | Gd-HP-DO3A | 3.8 | 0.3 | 0.999 | 3.9 | 0.3 | 0.999 |
| Dotarem® | Gd-DOTA | 3.7 | 0.3 | 0.999 | 3.7 | 0.3 | 0.999 |

**SuppTable 2:** Determination of the amount in mg/l of intact GBCA and the dissociated Gd(III)-ions in the presence of various $ZnCl_2$ and with (transchelation) or without (transmetallation) constant heparin (100 mM) concentrations.

**Transmetallation**

| $ZnCl_2$ | Omniscan® | | Optimark® | | Magnevist® | | MultiHance® | | Primovist® | |
|---|---|---|---|---|---|---|---|---|---|---|
| | Gd in solution | intact GBCA | Gd in solution | intact GBCA | Gd in solution | intact GBCA | Gd in solution | intact GBCA | Gd in solution | intact GBCA |
| mM | mg/l | | mg/l | | mg/l | | mg/l | | mg/l | |
| 0.125 | 0 | 27.88 | 0 | 27.88 | 0.47 | 27.4 | 0.29 | 27.59 | 0.14 | 27.74 |
| 0.25 | 0.12 | 27.75 | 0 | 27.88 | 0.93 | 26.95 | 0.93 | 26.95 | 0.37 | 27.51 |
| 0.5 | 0.03 | 27.85 | 0 | 27.88 | 1.73 | 26.15 | 1.43 | 26.45 | 1.18 | 26.7 |
| 1 | 0.42 | 27.46 | 0.3 | 27.57 | 3.08 | 24.79 | 3.12 | 24.76 | 2.34 | 25.53 |
| 2 | 0.76 | 27.11 | 0.72 | 27.16 | 5.4 | 22.48 | 5.93 | 21.94 | 4.83 | 23.05 |
| 4 | 1.97 | 25.91 | 1.81 | 26.07 | 9.05 | 18.83 | 10.12 | 17.75 | 8.36 | 19.52 |

**Transchelation**

| | Gd@ heparin | intact GBCA | Gd@ heparin | intact GBCA | Gd@ Heparin | intact GBCA | Gd@ heparin | intact GBCA | Gd@ heparin | intact GBCA |
|---|---|---|---|---|---|---|---|---|---|---|
| mM | mg/l | | mg/l | | mg/l | | mg/l | | mg/l | |
| 0.125 | 6.21 | 21.67 | 5.46 | 22.42 | 0.63 | 27.25 | 0.55 | 27.33 | 0.13 | 27.75 |
| 0.25 | 8.89 | 18.99 | 8.23 | 19.65 | 1.41 | 26.47 | 1.28 | 26.6 | 0.22 | 27.66 |
| 0.5 | 11.61 | 16.27 | 11.41 | 16.47 | 3.2 | 24.67 | 2.53 | 25.34 | 0.32 | 27.56 |
| 1 | 14.37 | 13.51 | 13.42 | 14.45 | 6.77 | 21.11 | 5.59 | 22.29 | 0.59 | 27.28 |
| 2 | 15.55 | 12.33 | 15.32 | 12.55 | 11.52 | 16.36 | 13.03 | 14.84 | 2.47 | 25.4 |
| 4 | 15.55 | 12.32 | 14.66 | 13.22 | 20.17 | 7.7 | 22.84 | 5.04 | 17.79 | 10.09 |

**SuppTable 3:** Table of the percentage contributions during the transmetallation in nano pure water as well as the transchelation in 100 µM heparin solution and constant 2 mM $ZnCl_2$ concentration.

| | water | GAG | GBCA | Gd in solution | |
|---|---|---|---|---|---|
| | % | | | | |
| Omniscan® | 37 | 0 | 57 | 5 | Transmetallation |
| Optimark® | 37 | 0 | 58 | 5 | |
| Magnevist® | 30 | 0 | 42 | 29 | |
| MultiHance® | 27 | 0 | 44 | 29 | |
| Primovist® | 27 | 0 | 62 | 11 | |
| Gadovist® | 35 | 0 | 65 | 0 | |
| ProHance® | 38 | 0 | 62 | 0 | |
| Dotarem® | 38 | 0 | 62 | 0 | |
| Omniscan® | 13 | 0 | 9 | 79 | Transchelation |
| Optimark® | 13 | 0 | 9 | 78 | |
| Magnevist® | 14 | 0 | 13 | 74 | |
| MultiHance® | 14 | 0 | 15 | 71 | |
| Primovist® | 23 | 0 | 53 | 23 | |
| Gadovist® | 33 | 0 | 62 | 5 | |
| ProHance® | 36 | 0 | 63 | 1 | |
| Dotarem® | 38 | 0 | 62 | 0 | |

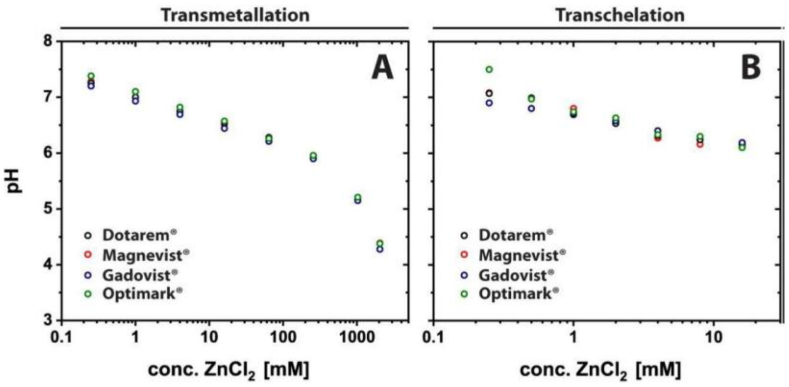


**Fig. S 1:** Representation of the influence of $ZnCl_2$ (0.125 – 2046 mM) on the pH values in solutions with Dotarem®, Magnevist®, Gadovist® and Optimark® in (A) milli-Q water and (B). 100 µM heparin solution. Decreasing pH values can be observed with increasing $ZnCl_2$ concentrations
